# Surgical Intervention for Adolescent Obesity: Evolution of the Scientific Agenda from 1980 to the Present

**DOI:** 10.1007/s11695-026-08486-8

**Published:** 2026-01-15

**Authors:** Ayşe Uçak, Fahriye Pazarcıkcı, Arzu Tat Çatal

**Affiliations:** 1 Faculty of Health Sciences, Department of Nursing, Burdur Mehmet Akif Ersoy University, Burdur, Turkey; 2 Distance Education Vocational School, Department of Medical Services and Techniques, Isparta University of Applied Sciences, Isparta, Turkey; 3 Faculty of Nursing, Akdeniz University, Antalya, Turkey

**Keywords:** Bariatric surgery, Adolescents, Obesity, Metabolic and bariatric surgery, Pediatric bariatric surgery

## Abstract

**Abstract:**

This study comprehensively analyzed scientific publications on metabolic and bariatric surgery in adolescents, using bibliometric methods to identify thematic trends and developments in the literature. Publications indexed in Web of Science, Scopus, and PubMed as of December 4, 2025, identified through the keyword combinations “adolescent” and “bariatric surgery,” were analyzed using Biblioshiny (Bibliometrix) and VOSviewer. The dataset included 2,174 articles published between 1980 and 2025, and 62% were published after 2016. *Obesity Surgery* was the leading journal, and the USA contributed the largest share of publications (42.1%). Thematic evolution analysis revealed a paradigm shift from bariatric surgery to metabolic and bariatric surgery. This reflects a transition from an early focus on surgical techniques and weight loss to broader issues such as metabolic mechanisms, psychosocial outcomes, pharmacotherapy integration, and health disparities. The study highlights the multidimensional development of adolescent bariatric surgery research and may inform future research and practice. Multidisciplinary approaches targeting holistic care and reducing global inequalities are recommended.

**Key Points:**

• The field has shifted from a technical focus on bariatric surgery to a holistic, biopsychosocial paradigm encompassing metabolic, psychological, pharmacotherapeutic, and social dimensions.

• The USA is the leading contributor to the field and dominates publication output, institutional productivity, and author influence.

• *Obesity Surgery Journal* is the most productive and influential journal and generates the highest volume of publications and citations.

## Introduction

Obesity is a significant public health problem affecting children and adolescents [1, 2]. More than 390 million children and adolescents aged 5–19 years are overweight, and 160 million of them are obese [1]. In the USA, one in five children is affected [3], while in Europe, one in three children (29% boys and 27% girls) has obesity [1, 2]. Rapid advancements in science and technology have transformed modern lifestyles and reduced daily physical activity, contributing to a sedentary pattern among children and adolescents. Furthermore, increased use of technological devices, electronic games, and the consumption of fast food and unhealthy snacks disrupt the balance between energy intake and expenditure, which increases the risk of obesity [4]. The growing prevalence of obesity has also led to higher rates of metabolic and associated cardiovascular problems, including type 2 diabetes, hypertension, dyslipidemia, and sleep apnea [5, 6]. Severe obesity during adolescence often persists into adulthood [5] and considerably increases lifelong morbidity and mortality [7]. Early and sustained weight control and metabolic improvement are, therefore, highly important [8].

Lifestyle modifications that include diet, exercise, and behavioral interventions are the first-line treatment for obesity; however, these are often insufficient in severe cases [9]. As a result, metabolic and bariatric surgery (MBS), long used in adults, has in recent years been considered an alternative option for children and adolescents [10, 11]. In adolescents, MBS may provide superior outcomes compared with conventional methods, especially in severe obesity and associated metabolic diseases [6]. An increasing number of studies have shown that MBS contributes to significant weight loss and remission of metabolic disorders in this age group [6, 12, 13]. The increasing use of sleeve gastrectomy, along with the introduction of robotic techniques and evidence on the effectiveness of Roux-en-Y gastric bypass (RYGB) for weight loss, reflects the evolution of surgical strategies [14, 15].

Although short- and long-term complications exist, many studies report that MBS is generally safe, well-tolerated, and effective in adolescents [16–18]. Despite the growing evidence, its application remains limited to a small proportion of adolescent patients [5, 19]. Moreover, access inequalities persist, especially among ethnic minorities and lower socioeconomic groups [20–23]. Therefore, careful patient selection, detailed planning, and long-term follow-up are required to achieve optimal outcomes [24]. For sustained weight control and metabolic improvement after MBS in adolescents, regular mental health monitoring, individualized support strategies, multidisciplinary care, structured daily routines, a supportive family environment, and consistent physical activity habits are essential [16].

Although scientific output on adolescent bariatric surgery has increased in recent years, the literature contains limited systematic, comprehensive, and quantitative analyses. A recent bibliometric review found that bariatric surgery publications largely focus on adults and that studies on adolescents are fewer in number and thematically fragmented [19]. Current evidence regarding long-term clinical outcomes, ethical considerations, psychosocial outcomes, and standardized surgical indications for adolescents is also limited and heterogeneous [25]. This is attributed to the dependence of treatment protocols on adult data, even though adolescents differ from adults in physiological and psychosocial development [26]. As a result, the current knowledge base on adolescent bariatric surgery is insufficient to guide evidence-based clinical practice and support multidisciplinary decision-making processes [27]. These gaps highlight the need for a bibliometric evaluation that can identify thematic evolution, key themes, productive institutions, and international collaborations. The findings of this analysis may increase the visibility of academic output and contribute to the direction of future research and health policy [28]. By identifying research gaps and existing knowledge networks, the findings may also provide a guiding framework for future studies.

## Methods

### Research Purpose and Questions

The aim of this study was to analyze the development of the scientific literature on adolescent MBS over the past 45 years. Bibliometric methods were used to identify publication trends, key research themes, collaborations, and gaps in the literature. The study sought to address the following questions:

1. What is the distribution of academic publications and citations on adolescent MBS over the years?
2. Which journals, countries, institutions, and authors have been the most productive in this field?
3. According to citation analyses, which studies have been the most influential?
4. How are current collaboration networks structured?
5. What key concepts and thematic focuses appear in the literature, and which research gaps warrant further investigation?

### Design

This study examined the development of scientific publications on adolescent MBS from a bibliometric perspective. The research design, data management, and reporting followed the BIBLIO checklist [29], which was developed for bibliometric reviews and is supported by EQUATOR.

## Data Collection

**Fig. 1:**
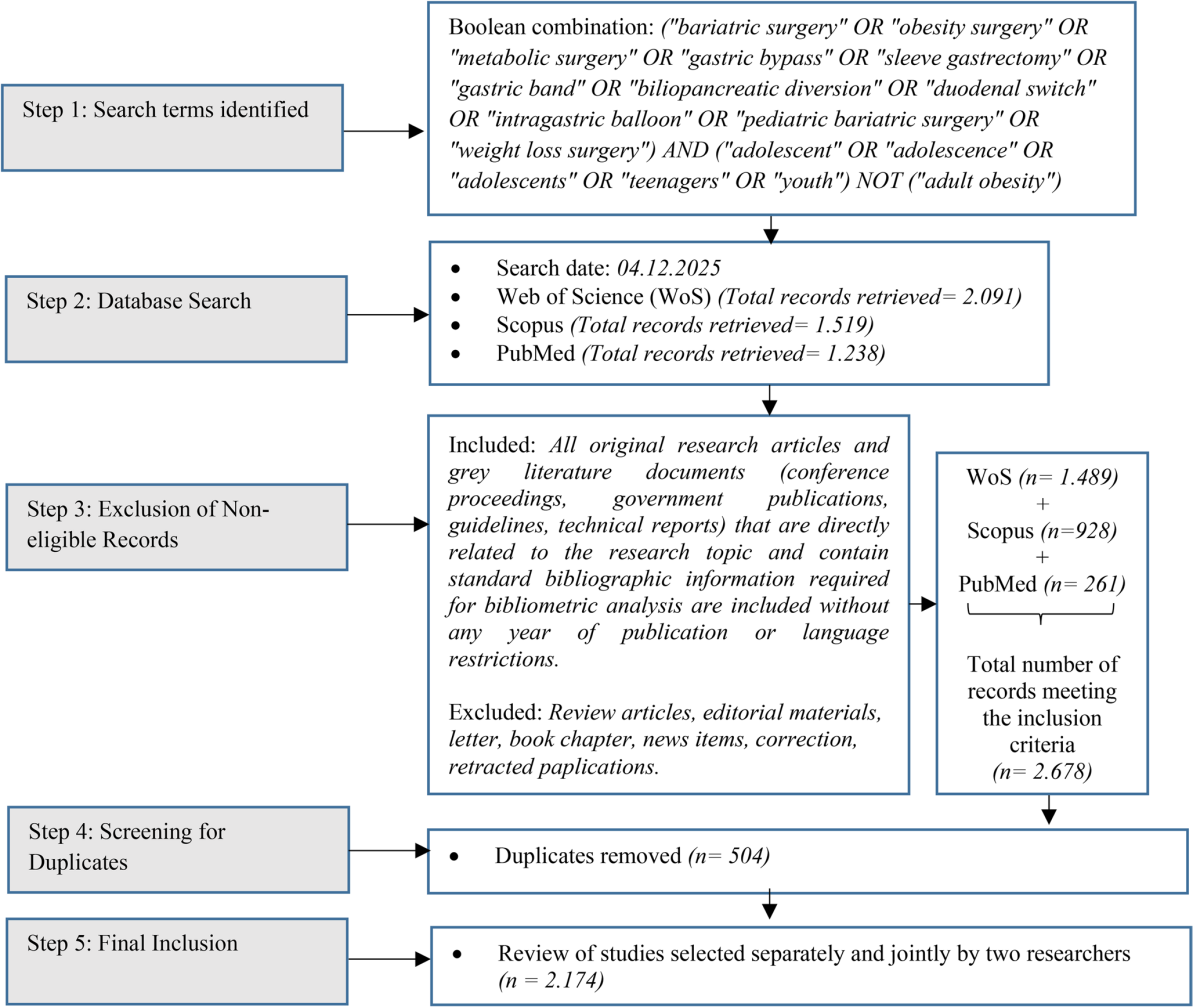
PRISMA diagram developed by the authors

A five-step publication selection process was followed to ensure accuracy in screening the relevant literature (Fig. 1). First, a search strategy was developed using combinations of the keywords “adolescent” and “bariatric surgery” with Boolean operators. Second, Web of Science (WoS), Scopus, and PubMed, widely used in health sciences and interdisciplinary research, were selected to provide comprehensive coverage of global academic output [30]. Third, a search was conducted on December 4, 2025, across titles, abstracts, and keywords in WoS, Scopus, and PubMed, and no year or language restrictions were applied. Records identified through this search were screened using predefined inclusion criteria (*n* = 2,678). Fourth, duplicate records were removed (*n* = 504). In the final stage, studies that met the inclusion criteria were reviewed separately and then jointly by two researchers (*n* = 2,174).

### Inclusion and Exclusion Criteria

All original research articles and gray literature documents (conference proceedings, government publications, guidelines, technical reports) that were directly related to the research topic and contained the bibliographic information required for bibliometric analysis were included without year or language restrictions. To ensure analytical integrity, review articles, editorial materials, letters, book chapters, news items, corrections, and retracted publications were excluded. Additionally, duplicate records were removed (Fig. 1).

### Bibliometric Method

Two software programs were selected for bibliometric visualization and analysis: the Biblioshiny interface of Bibliometrix and VOSviewer. Bibliometrix provides interactive analysis and flexible data management within an R-based environment [31], and VOSviewer is a powerful and widely used tool for visualizing scientific relationship networks such as authors, institutions, keywords, and citations [32]. Using both tools enabled a multifaceted, detailed analysis of bibliometric datasets from different methodological perspectives [32, 33]. Within the scope of the analysis, publication and citation years; citation patterns; and the distribution of publications by journals, research areas, and countries were evaluated.

### Ethical Considerations

This study used only publicly available and secondary bibliometric data. The dataset contained no personal or confidential information and was obtained from open-access publication, author, institution, and citation records. No procedures were performed that could reveal personal information, and the findings were interpreted solely for scholarly purposes.

## Results

### General Information

This bibliometric study analyzed 2,174 publications on adolescents who underwent bariatric surgery, published between 1980 and 2025 and meeting the inclusion criteria. These studies appeared in 702 sources and were authored by 8,330 researchers. The average number of co-authors per article was 6.35. A total of 117 were single-authored, and 9.11% involved international collaboration. The average number of citations per article was 26.04. In total, 2,713 keywords were used, and the average document age was 8.37 years.

### Publication and Citation Numbers by Year

**Fig. 2:**
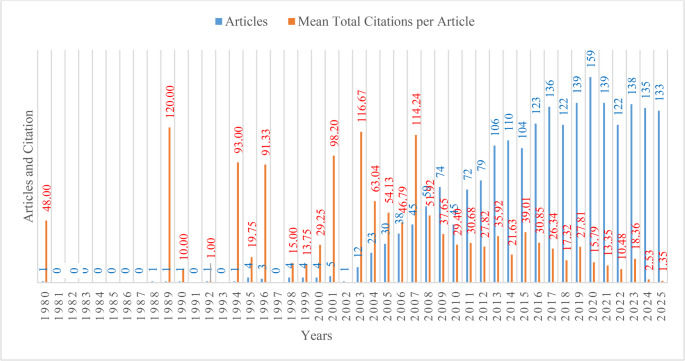
Annual trends in publication output and mean citations per article (1980–2025)

Examining changes in publication volume helps reveal shifts in research trends and focal points over time [34]. Figure 2 shows the annual distribution of the 2,174 studies published between 1980 and 2025, as well as annual trends in average citation counts per article. Although only a limited number of studies were published between 1980 and 2005, publication output increased after 2005 and gained momentum from 2012. After 2016, 62% of the articles were published, the annual number of publications exceeded 120, and the highest value was recorded in 2020 with 159 articles. In the first eleven months of 2025, 133 articles were published. The annual growth rate, calculated using Bibliometrix software, was 11.48%. To confirm the trend statistically, a log-linear regression model was applied, and the annual growth rate was calculated as 16.83% (95% confidence interval [14.4–19.3], *p* < 0.001). Both approaches supported the upward trend in publications.

Citation counts are a key indicator of scientific impact and reflect the visibility and influence of published studies [35]. Figure 2 shows that 1989 was the peak year for average citations, reaching 120. The high average citation values in 2003 and 2007 (114.24 and 116.67, respectively) also indicate strong influence during those years. After 2016, average citation counts trended downward.

### Journal Analysis

**Fig. 3:**
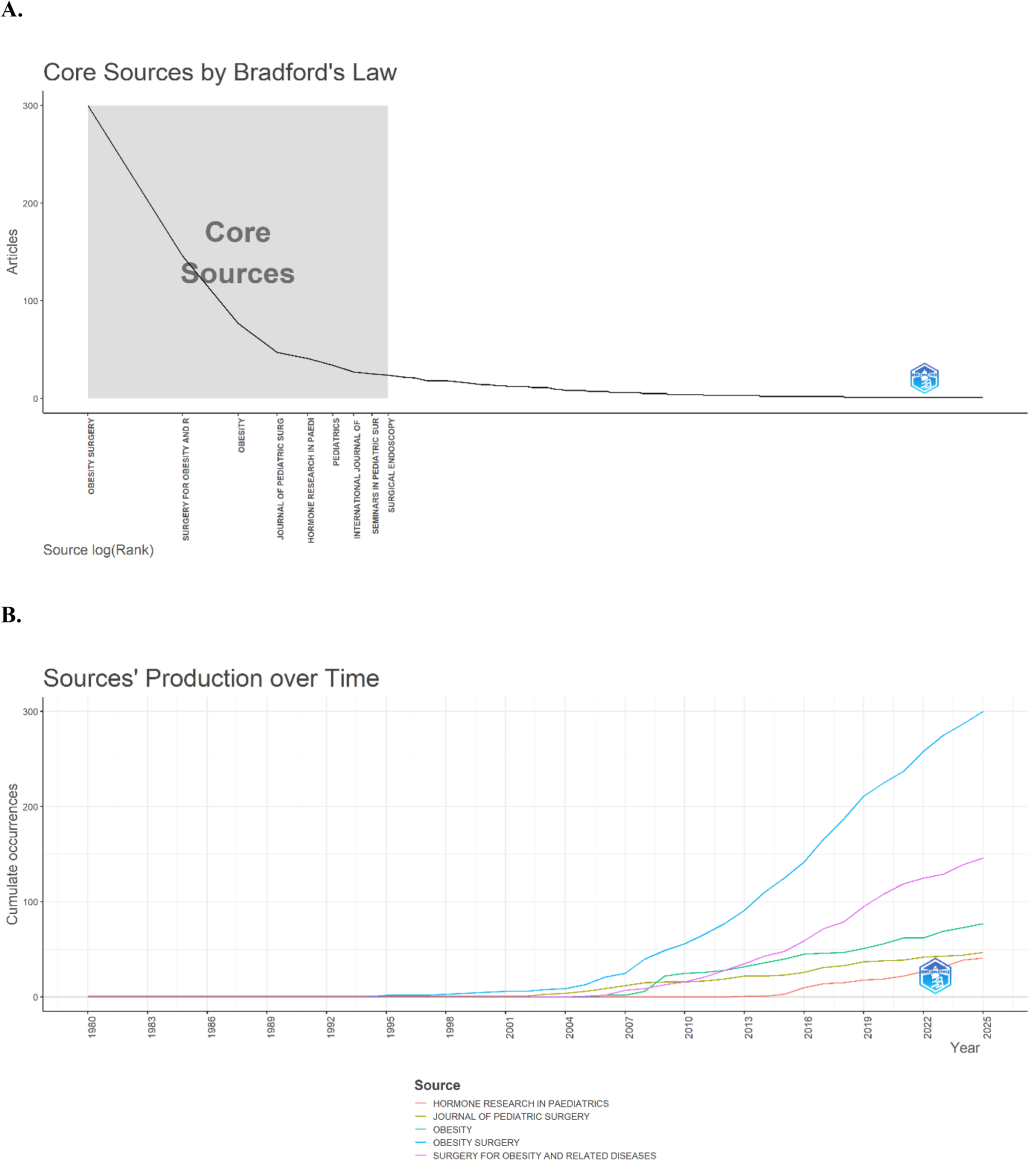
**A** Core sources by Bradford’s Law, **B** Sources production over time

Identifying the most productive and influential academic journals in this field is important for researchers in terms of both literature reviews and publication strategies [34]. The journals that contributed most to this topic and were most influential are shown in Fig. 3A. According to the analysis conducted with Biblioshiny, 34.18% of the total 2,174 articles were published in the top ten journals, indicating their central position in the field’s literature. Two journals stood out in this analysis. *Obesity Surgery* was the most productive and influential journal, with 300 (13.80%) publications, and *Surgery for Obesity and Related Diseases* ranked second, with 146 (6.71%) publications. The local impact index (H) was 30 for *Obesity Surgery* and 29 for *Surgery for Obesity and Related Diseases*. The graph titled “Production of Sources Over Time” in Fig. 3B shows the cumulative productivity of journals with the highest number of publications in this field. Contributions to journals were limited until the early 2000 s, but a marked upward trend was observed after 2005. Among the journals examined, *Obesity Surgery* stood out by far and showed a rapid increase in publications after 2010 (Fig. 3B).

### Country and Institutions Analysis

Analyzing publication data by country helps determine each country’s research contributions and evaluate international collaboration (34). The USA had the highest number of publications (*n* = 916) and citations (TC = 32.331; average citations per article = 35.30). A total of 42.1% of all articles were published in the USA. Of these, 859 articles were written solely by researchers based in the USA, and 57 articles resulted from collaborations between the USA and other countries. International collaborations occurred in 6.2% of USA-based studies. Germany (63 articles) and Italy (59 articles) followed, while Türkiye ranked 16th with 20 publications.

In total citation count, the UK (TC = 2.451; average citations per article = 58.40) and Italy (TC = 1.760; average citations per article = 29.80) ranked second and third, respectively. Despite its low publication volume, Türkiye ranked as the third most influential country, with 1.154 citations and an average of 57.70 citations per article. The institution with the most relevant publications was Cincinnati Children’s Hospital Medical Center, with 338 publications, followed by the University System of Ohio and Harvard University (Table 1).

**Table 1:** Analysis of publication and citation data by country, and organization

Country	Articles	Articles %	SCP	MCP	MCP %	Country	TC	Average Article Citations	Organization	Articles
United States	916	42.1	859	57	6.2	United States	32,331	35.30	Cincinnati Children’s Hospital Medical Center	338
Germany	63	2.9	51	12	19	United Kingdom	2451	58.40	University System of Ohio	293
Italy	59	2.7	52	7	11.9	Italy	1760	29.80	Harvard University	292
Sweden	50	2.3	47	3	6	Germany	1463	23.20	Harvard University Medical Affiliates	221
Israel	48	2.2	43	5	10.4	Canada	1431	32.50	Massachusetts General Hospital	188
Brazil	45	2.1	31	14	31.1	Australia	1334	38.10	Ohio State University	143
France	44	2	39	5	11.4	Sweden	1295	28.80	University of Cincinnati	134
United Kingdom	42	1.9	30	12	28.6	Türkiye	1154	57.70	University of Colorado System	125
Canada	41	1.9	36	5	12.2	Greece	961	160.20	University of Colorado Anschutz Medical Campus	115
Saudi Arabia	39	1.8	37	2	5.1	China	705	14.10	Harvard Medical School	106

Note **:** *SCP* Single Country Publications, *MCP* Multiple Country Publications, *TC* Total Citations

### Author Performance Analysis

At the center of the network was Thomas H. Inge, with 201 publications and 34.29 fractionalized publications. He was followed by Todd M. Jenkins, Marc P. Michalsky, and Meg H Zeller. These four authors formed the intellectual core of the network and played a central role in shaping the scientific direction of the field (Table 2).

**Table 2:** Author performance analysis

Author	Articles	Fractionalized publications
Inge, Thomas H	201	34.29
Jenkins, Todd M	114	13.40
Michalsky, Marc P	72	8.73
Zeller, Meg H	68	10.47
Xanthakos, Stavra A	52	7.23
Courcoulas, Anita P	48	4.19
Helmrath, M. A	48	5.27
Nadler, E	44	8.98
Olbers, Torsten	43	5.97
Misra, M	41	5.73

**Fig. 4:**
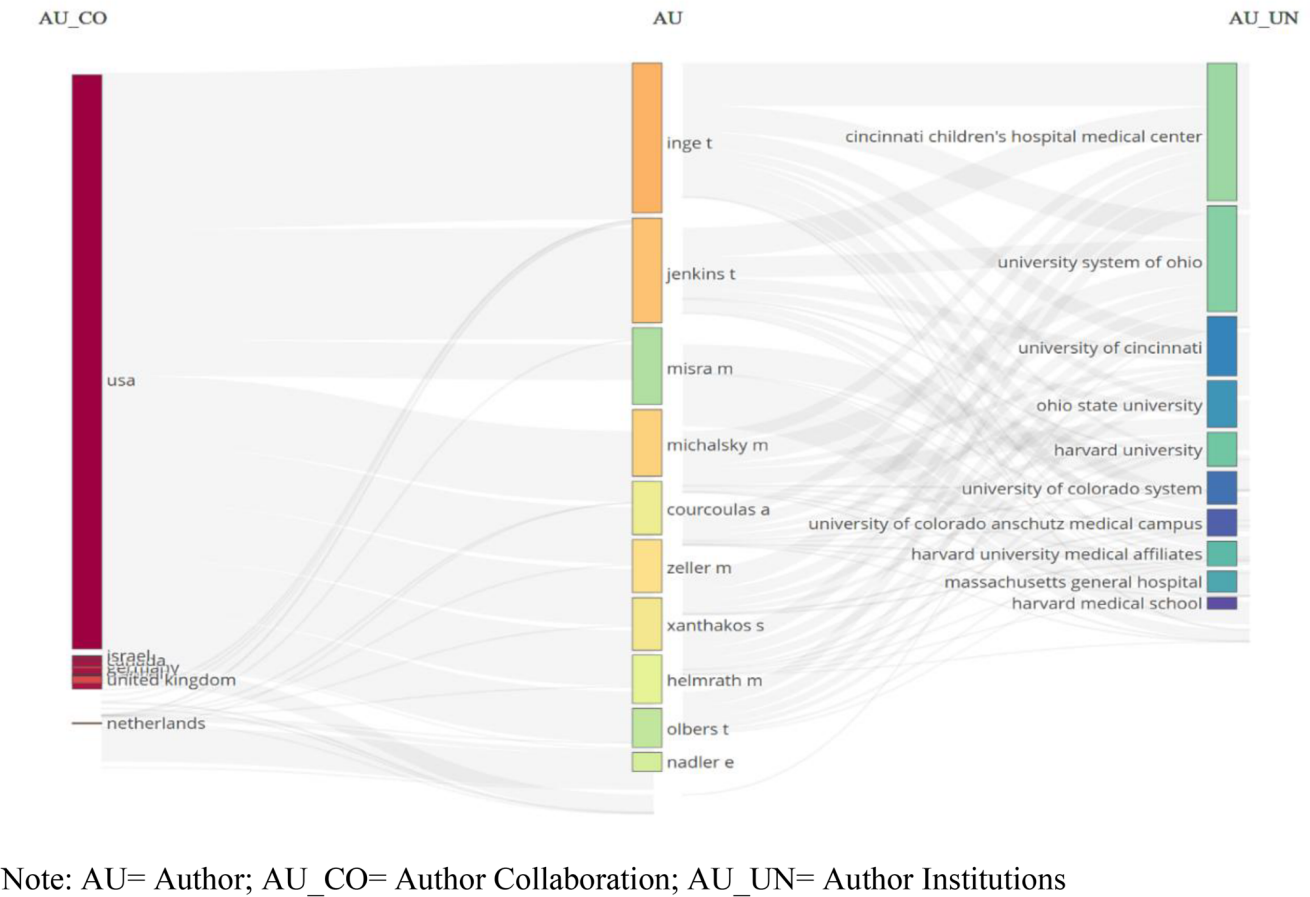
Author, country, and organization collaboration shown in a three-field plot

The three-field plot in Fig. 4 illustrates the relationships among authors, their affiliated institutions, and the countries with which they collaborated. The field’s principal researchers were based in the USA (Thomas H. Inge, Todd M. Jenkins, Marc P. Michalsky, and Madhusmita Misra), the most productive institutions were located in the USA (Cincinnati Children’s Hospital Medical Center, University System of Ohio, and Harvard University), and international collaboration spread largely from the USA to Europe (the UK, Italy, Israel, and the Netherlands) (Fig. 4).

### Document Analysis

The top ten academic studies with the highest global citations in the field of adolescent obesity and surgery are presented in Table 3. The evaluation considered total citations, average annual citations, and normalized citation counts. The study by Barlow SE, published in *Pediatrics* in 2007, was by far the most cited and remains highly influential, with 3,392 citations and an average of 178.53 citations per year. The study by Yumuk and colleagues, published in 2015 and cited 987 times, was the second most influential publication (annual average citations = 89.73; normalized TC = 25.30). The study by Hampl et al. [36], also published in *Pediatrics*, had the highest average impact score despite being recent (annual average citations = 314.38; normalized TC = 51.38). Studies by Ludvigsson et al. [37] and Styn et al. [38] were other important publications notable for their normalized impact scores (30.20 and 31.59, respectively) (Table 3).

**Table 3:** Most global cited documents

Paper	DOI	Total Citations	TC per Year	Normalized TC
Barlow S, 2007, Pediatrics	10.1542/peds.2007-2329C	3392	178.53	29.69
Yumuk V, 2015, Obesity Facts	10.1159/000442721	987	89.73	25.30
Hampl S, 2023, Pediatrics	10.1542/peds.2022-060640	943	314.33	51.38
Ludvigsson J, 2019, Eur J Epidemiol	10.1007/s10654-019-00511-8	840	120.00	30.20
Styne D, 2017, J Clin Endocr Metab	10.1210/jc.2016-2573	832	92.44	31.59
Kelly A, 2013, Circulation	10.1161/CIR.0b013e3182a5cfb3	771	59.31	21.47
Kaditis A, 2016, Eur Respir J	10.1183/13993003.00385-2015.00385	706	70.60	22.89
Welbourn R, 2019, Obes Surg	10.1007/s11695-018-3593-1	649	92.71	23.33
Williams E, 2015, Curr Obes Rep	10.1007/s13679-015-0169-4	639	58.09	16.38
Stunkard A, 2003, Biol Psychiat	10.1016/S0006-3223(03)00608-5	576	25.04	4.94

Note: *TC* Total Citations

### Key Concepts and Thematic Foci Analysis

**Fig. 5:**
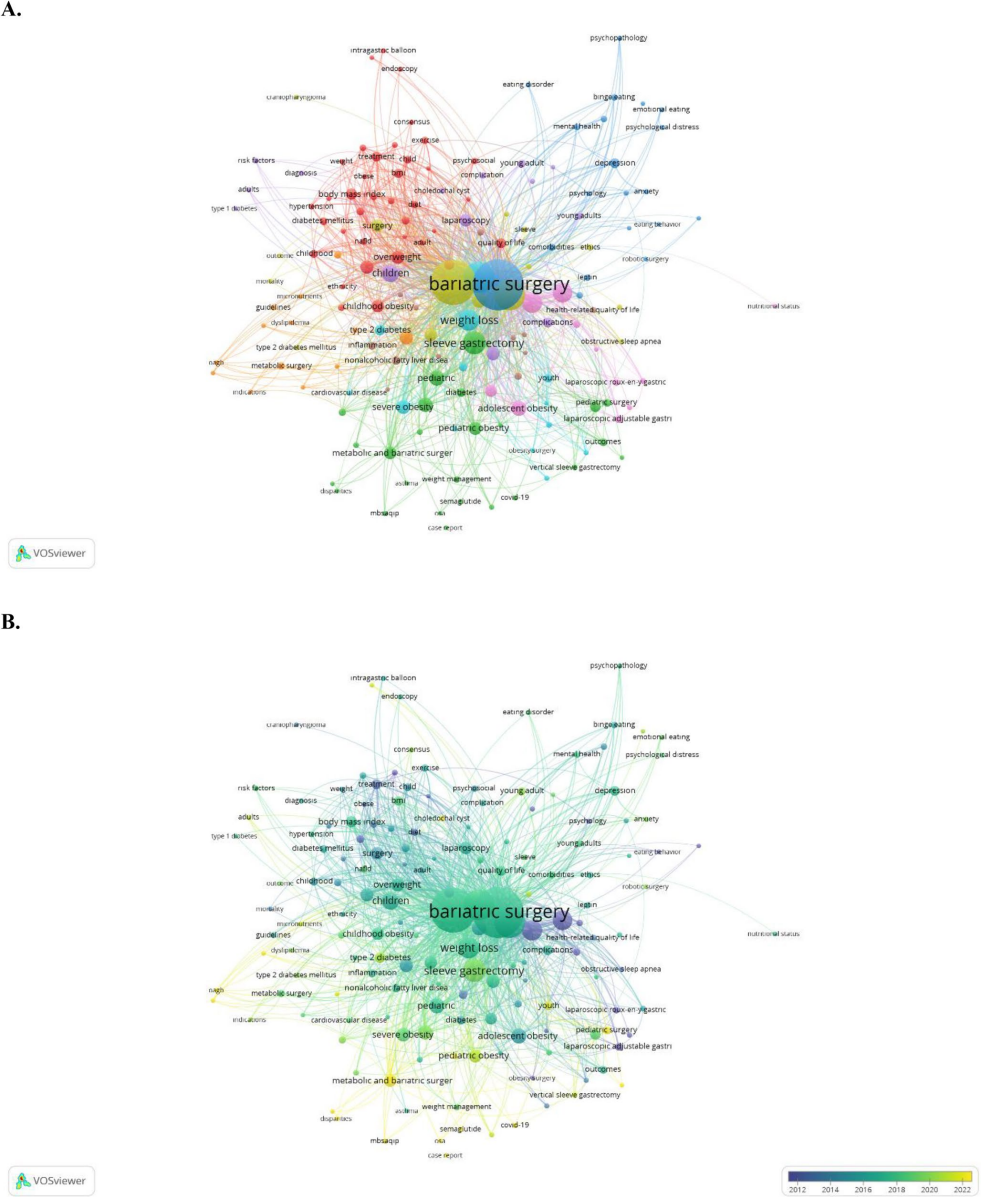

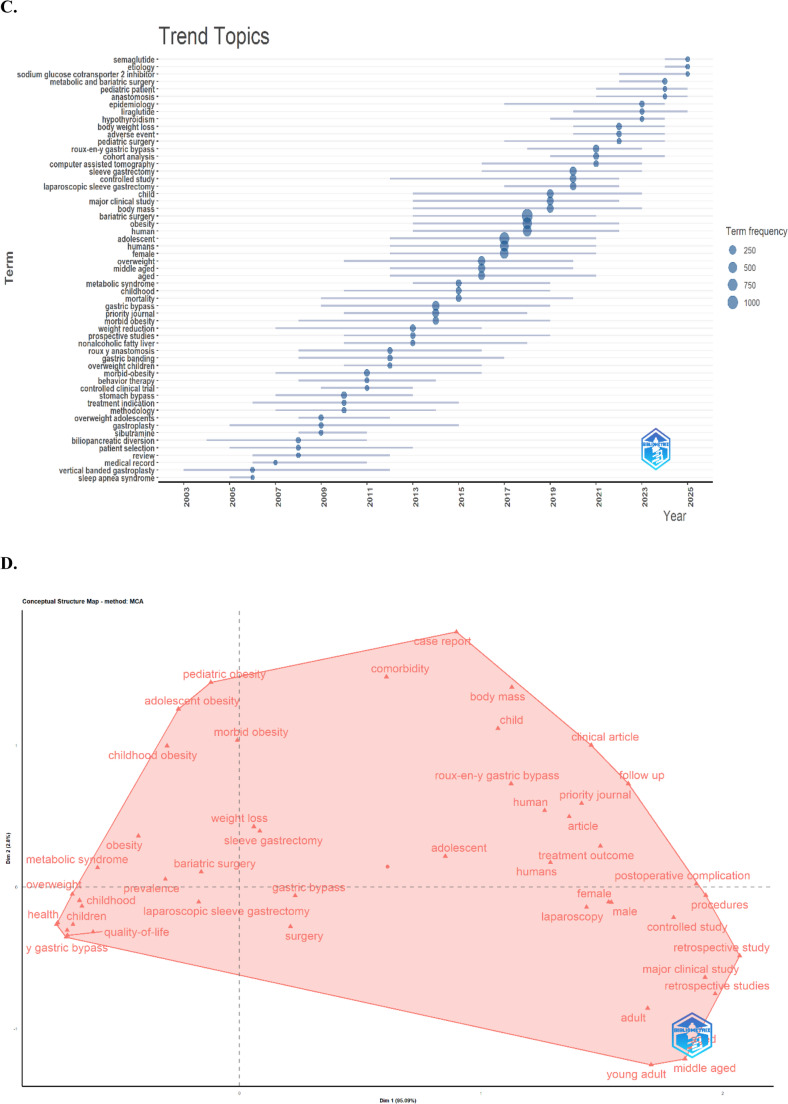
**A** Analysis of the co-occurrence of keywords-Visualization, **B** Analysis of the time-based co-occurrenceof keyword-Visualization **C** Trends over the years, **D** Factorial Analysis

Co-word analysis is an important bibliometric method for revealing the conceptual structure of studies in the literature, identifying the most prominent and current topics in the field, and systematically evaluating the relationships among these topics [19]. The keyword co-occurrence network in Fig. 5A illustrates the conceptual structure of research themes within the field of bariatric surgery. Of the 2,713 keywords identified, 160 terms that co-occurred at least five times were included in the analysis. The network contained nine conceptual clusters, 2,165 links, and a total link strength of 5,421. The most central concept in the network was “bariatric surgery” (*n* = 524), around which highly connected terms such as “obesity” (*n* = 427), “adolescents” (*n* = 395), “sleeve gastrectomy” (*n* = 113), and “weight loss” (*n* = 90) were positioned. The overall network structure showed that bariatric surgery formed a multidimensional research domain that encompassed a broad thematic spectrum ranging from surgical techniques to psychosocial outcomes and from metabolic disorders to childhood obesity (Fig. 5A).

The time-based keyword co-occurrence map (Fig. 5B) illustrates the thematic evolution within the field. According to the color scale, blue tones represent early-period research topics, green indicates mid-period studies, and yellow denotes current research trends. In the early period (2012–2015), core clinical and methodological themes such as “bariatric surgery,” “obesity,” “gastric bypass,” and “weight loss” dominated the literature. In the mid-period (2016–2019), themes such as “pediatric and adolescent obesity,” “laparoscopic sleeve gastrectomy,” “type 2 diabetes mellitus,” and “quality of life” appeared more frequently. Current keywords (2020–2025) reflect the emerging directions of the field. Among them, “semaglutide,” “covid-19,” “metabolic and bariatric surgery,” “disparities,” and “weight management” were notable (Fig. 5B).

In the trend topic analysis presented in Fig. 5C, the keywords most frequently used in the studies showed a distinct distribution over time. In the early period (2005–2010), terms such as “sleep apnea syndrome,” “vertical banded gastroplasty,” “medical record,” “review,” “patient selection,” “biliopancreatic diversion,” and “gastritis” were prominent. Since 2010, concepts such as “overweight adolescents,” “treatment indications,” “controlled clinical trials,” “gastric bypass,” “morbid obesity,” “gastric banding,” “Roux-en-Y anastomosis,” “non-alcoholic fatty liver disease,” and “prospective studies” have gained importance. Between 2013 and 2018, terms such as “weight loss,” “adolescent obesity,” “gastric bypass,” “childhood,” “metabolic syndrome,” “middle-aged,” “women,” “people,” “adolescents,” “obesity,” “bariatric surgery,” and “body mass index” were used with high frequency. In recent years (2018–2024), terms such as “large clinical trials,” “children,” “laparoscopic sleeve gastrectomy,” “gastric sleeve,” “cohort analysis,” “computed tomography,” “Roux-en-Y gastric bypass,” “serious events,” “pediatric surgery,” “body weight loss,” “hyperinsulinism,” “hypertension,” “epidemiology,” “anastomosis,” “pediatric patients,” “metabolic and bariatric surgery,” “sodium-glucose cotransporter 2 inhibitors,” and “semaglutide” have been increasingly emphasized (Fig. 5C).

Multidimensional scaling (MDS) was applied to analyze the structural relationships among key concepts. MDS visualizes conceptual similarities in a two-dimensional plane, allowing conceptual clustering and separation to be observed. In the MDS map in Fig. 5D, the horizontal axis (Dim 1) explained 95.09% of the total variance, and the vertical axis (Dim 2) explained 2.08%. Together, the two dimensions accounted for 97.09% of the variance. The first dimension distinguished studies that focused on clinical and surgical characteristics (e.g., laparoscopy, gastric bypass, treatment outcome, and postoperative complications) from studies that emphasized pediatric and metabolic perspectives. The second dimension reflected a gradient between adult-focused clinical interventions and childhood or adolescent obesity research. Central concepts, including bariatric surgery, sleeve gastrectomy, and weight loss, were positioned near the intersection of both dimensions, indicating their integrative role across thematic clusters. The left cluster grouped terms such as pediatric obesity, childhood obesity, metabolic syndrome, and quality of life, representing research on metabolic and psychosocial outcomes in younger populations. The right cluster included terms such as laparoscopy, controlled study, and retrospective study, reflecting methodologically oriented adult clinical research. Overall, the MDS results showed that bariatric surgery literature has evolved into a multidimensional field that spans clinical, metabolic, and demographic domains (Fig. 5D).

## Discussion

This study evaluated the literature on bariatric surgery in adolescents from a multidimensional perspective using bibliometric methods. It addressed how developments in the literature have evolved over time, which themes have become central, and how academic productivity has been distributed geographically. Although this study included articles published since 1980 [39], reports of successful open RYGB procedures exist from earlier periods [40]. With the widespread adoption of laparoscopic surgical techniques in the 2000 s, the literature underwent a significant transformation [17]. Since the mid-2000s, the initiation of large-scale, prospective cohort studies such as Teen-LABS (Teen-Longitudinal Assessment of Bariatric Surgery) has enhanced the scientific legitimacy of adolescent bariatric surgery and attracted greater interest from researchers [7]. The publication of clinical guidelines for pediatric and adolescent bariatric surgery by the American Society for Metabolic and Bariatric Surgery and other professional associations further increased standardization and clarified research priorities [41]. The global rise in pediatric obesity and growing awareness of its long-term health consequences have also strengthened academic interest [42]. Lister et al. [43] reported that child and adolescent obesity is high in many high-income countries and continues to rise in low- and middle-income countries. They also found that severe obesity is more likely to occur in adolescents and is linked to a reduced quality of life. In the USA, it is estimated that by 2050, 43.1 million children and adolescents aged 5–24 will be overweight or obese [44]. Recent publications also note that the prevalence of adolescent obesity is increasing in the USA and that the number of young people who meet the criteria for advanced interventions such as MBS is expanding [4, 45, 46]. The global rise in childhood and adolescent obesity rates [47] highlights the scientific importance of this issue and supports the growth of the literature.

In line with these observations, this study found that the adolescent bariatric surgery literature showed an exponential growth rate of 11.48–16.83% annually. This rate indicates that the field is rapidly developing and dynamic. Although data for 2025 were not yet complete, the 133 articles published in the first eleven months of the year reflect the growing scientific importance of bariatric surgery in the context of adolescent obesity and suggest that the field is maturing academically and deepening thematically. These projections highlight the increasing seriousness of adolescent obesity and indicate that research in this field will continue to deepen and diversify.

Our bibliometric analyses revealed that the journals *Obesity Surgery* and *Surgery for Obesity and Related Diseases* held a central position in terms of publication numbers and citation rates. These findings are consistent with those of studies by Seçkin and Cebeci (2024) [19], Domínguez Alvarado et al. (2024) [48], Corrêa et al. (2024) [49], and Tunç Tuna and Uslu (2025) [50]. These journals enhance the academic visibility of adolescent bariatric surgery and shape scientific directions in the field. Bradford (1985) [51] noted that publication concentration is not a random phenomenon but rather an indication that a field has reached a stage of scientific maturation or disciplinary centralization. In the present study, the clustering of research on adolescent MBS within these two journals may similarly be interpreted as evidence of such maturation. However, this consolidation may also introduce a potential limitation with respect to publication diversity. The numerical predominance of articles appearing in leading journals may reduce the visibility of research published in smaller or newly emerging journals.

Geographical and institutional analyses showed that the USA dominated the literature in terms of scientific productivity and impact, consistent with previous studies [44, 48–50]. Recent narrative and systematic reviews further demonstrate that, when performed in appropriate centers, adolescent MBS yields substantial and durable weight loss, leads to significant improvement or remission of associated cardiometabolic problems, and has a safety profile comparable to that observed in adults [45, 46, 52]. Till et al. [53] also recommended high-volume multidisciplinary centers and long-term follow-up for adolescents undergoing MBS. The pronounced dominance of the USA in this field may be explained by several structural factors. These include the high prevalence of obesity in both adults and adolescents in the country, the long-standing presence of high-volume and specialized pediatric obesity centers, and the availability of multidisciplinary MBS teams. These well-established centers enable large-scale, long-term prospective studies, which are further supported by robust research funding mechanisms and a strong scientific infrastructure. Furthermore, the effectiveness of this study in European countries such as the UK, Italy, Israel, the Netherlands, and Türkiye demonstrates the growing acceptance of pediatric bariatric surgery on the continent.

Author analysis showed that the most productive and influential author was Thomas H. Inge, followed by Todd M. Jenkins, Marc P. Michalsky, and Meg H. Zeller. These authors formed the intellectual core of the network in terms of productivity and international collaborations, maintaining strong academic ties with the USA, the UK, Israel, and the Netherlands. The literature also indicates that while international collaborations exist among countries in North America, Europe, and Asia, cooperation among Latin American countries is limited [48]. However, international collaboration in this field appears to be generally low for a global health issue. Although the low rate of collaboration in bariatric surgery research aligns with the field’s clinical orientation and operational independence, MBS represents shared global health problems. Strengthening international collaborative networks is, therefore, considered important for scientific advancement. Furthermore, the present study’s finding that single-authored articles were rare supports the view that the field relies on teamwork rather than individual efforts. This is consistent with recent publications emphasizing the need for a multidisciplinary approach to patient care [45, 46, 52, 54].

The results of this study showed that the early literature in the field was dominated by basic surgical and clinical concepts such as bariatric surgery, gastric bypass, obesity, and weight loss. This early body of work also focused on the feasibility, safety, and short-term weight loss outcomes of surgical techniques. However, keyword trends indicate that the field has undergone a paradigm shift from “bariatric surgery” to “metabolic and bariatric surgery” and moved toward a biopsychosocial approach. The early focus on surgical technique and weight loss gave way to broader topics such as metabolic mechanisms, psychosocial outcomes, pharmacotherapy integration, and health disparities. As Corrêa et al. [49] noted, the emergence of different focal points, such as surgical interventions, clinical case management, basic research, body contouring, and surgical risk studies, is consistent with these results. These findings align with current trends in the literature on adolescent bariatric surgery, which shows that studies have long focused mainly on surgical procedures, weight loss, and metabolic outcomes [24, 55–57].

Some studies indicate a significant gap in the literature on MBS regarding the exploration of psychosocial dimensions [49, 55]. It is emphasized that bariatric surgery should be considered not only in terms of traditional outcomes such as weight loss or surgical success but also in relation to psychological well-being, quality of life, and long-term behavioral outcomes. Although improvements in depression and anxiety symptoms following bariatric surgery have been reported, these improvements are not always permanent, vary between individuals, and often involve a psychologically complex process [55]. The importance of integrating psychological support services into postoperative follow-up programs has also been noted [56]. These findings highlight the need to standardize preoperative and postoperative psychological assessments and to adopt patient-centered, multidisciplinary approaches. As such, sustainable psychosocial well-being can be promoted alongside weight loss.

Health-related quality of life is a well-established research area in bariatric surgery [50]. MBS is known to have positive effects on adolescents, especially in improving body image, social relationships, and self-esteem [50, 55, 57]. These findings are also in line with our multidimensional scaling analysis, which showed that the concept of “quality of life” clustered closely with “bariatric surgery,” “adolescents,” and “weight loss.” Thus, quality of life constitutes an important component of overall surgical outcomes beyond physical changes.

The study also identified research gaps that warrant further investigation. The prominence of studies on topics such as semaglutide and sodium-glucose cotransporter 2 inhibitors suggests that the use of GLP-1 receptor agonists and other anti-obesity medications alongside bariatric surgery has become an active area of ​​research [58]. This trend suggests that future research may place greater emphasis on pharmacotherapy. The prominence of the term “disparities” reflects the growing attention to socioeconomic, racial, and geographic inequalities in access to healthcare [59]. This theme emphasizes that pediatric bariatric surgery is not only a clinical issue but also a health equity issue. The emphasis on the term “weight management” suggests that long-term postoperative weight management and the prevention of weight gain are important research priorities [60]. The median documented age of 8.37 years indicates that long-term follow-up data are limited. Studies examining the 20- to 30-year outcomes of adolescent surgery are needed [61]. The low rate of international collaboration shows the potential for establishing stronger research networks, particularly between the USA and Europe. The prominence of themes such as quality of life, mental health, and psychosocial outcomes necessitates further research in these areas [62]. Developing predictive models that identify which patients benefit most from specific procedures is another important direction for future research [26].

### Limitations

Despite its strengths, this study has several limitations. The first limitation relates to the nature of bibliometric analysis. In this study, all publications were evaluated equally, regardless of their methodological quality. The coverage of the databases used (WoS, Scopus, and PubMed) may have excluded some journals or conference proceedings. The time required for new publications to accumulate citations may have underestimated the impact of recent studies. Furthermore, differences in terminology across authors may also have influenced the conceptual analysis. Additionally, certain limitations in keyword selection may have led to the exclusion of some studies from the analysis.

## Conclusion

This study provides a valuable and comprehensive scientific foundation for researchers, clinicians, and policymakers by systematically and multidimensionally outlining the historical, thematic, and geographical development of the adolescent MBS literature. The findings identify gaps in the current literature and may inform future research directions. To address the obesity epidemic, efforts should focus on reducing global inequalities in access to MBS, enhancing clinical practices through the effective use of robotics and artificial intelligence, informing policy development, and improving patient quality of life.

## Data Availability

All data generated or analysed during this study are included in this article. The datasets used during the current study are available from the corresponding author on reasonable request.
